# Simplified Laboratory Cultivation of *Thiobacillus denitrificans* and *Sulfurimonas denitrificans* with Practical Guidelines for Laboratories Without Advanced Anaerobic Equipment

**DOI:** 10.3390/microorganisms14081711

**Published:** 2026-08-04

**Authors:** Barbara Kalebic, Zvonimir Prgic, Aleksandra Marsavelski, Tomislav Ivankovic

**Affiliations:** 1 Agrai Biotech d.o.o., Matekov Brijeg 10, Brežane Lekeničke, 44272 Lekenik, Croatia; kalebicbarbara@gmail.com; 2 Department of Biology, Faculty of Science, University of Zagreb, Horvatovac 102A, 10000 Zagreb, Croatia; zvone3prgic@gmail.com; 3 Department of Chemistry, Faculty of Science, University of Zagreb, Horvatovac 102A, 10000 Zagreb, Croatia

**Keywords:** anaerobic digestion, biogas, anaerobic cultivation, microscopy techniques

## Abstract

Chemolithotrophic denitrifying bacteria *Thiobacillus denitrificans* and *Sulfurimonas denitrificans* are model organisms for sulfur and nitrogen cycling and are frequently used as model organisms in biogeochemical and applied research. Their cultivation is considered technically demanding, largely due to the assumption that strict anaerobic equipment is required. Here, we provide a simplified, reproducible cultivation workflow designed for laboratories with limited anaerobic infrastructure, with a special focus on establishing growth under practical pseudo-anoxic conditions. Three media were evaluated (modified DSMZ 113, ATCC 152 *Thiobacillus* broth, and a commercial *Thiobacillus* broth) in both liquid and solid formats using anaerobic jars and a sealed-bottle “pseudo-anoxic” approach. Consistent growth of both organisms was achieved exclusively in modified DSMZ 113. Importantly, *T. denitrificans* grew reproducibly not only under anaerobic jar conditions but also under the sealed-bottle pseudo-anoxic setup, demonstrating that successful cultivation is feasible without specialized anaerobic systems. The *S. denitrificans* grew successfully, but exclusively under anoxic conditions. Additionally, we describe a simple and novel coverslip-on-agar microscopy method for rapid confirmation of small transparent colonies, supporting reliable isolation and contamination control. Overall, our results provide a practical step-by-step cultivation guide, highlighting that *T. denitrificans* can be robustly cultured even without complying with strict protocol for anaerobic cultivation.

## 1. Introduction

Chemolithotrophic denitrifying bacteria, particularly *Thiobacillus denitrificans* and *Sulfurimonas denitrificans*, occupy an important ecological niche in sulfur and nitrogen cycles [1]. These bacteria oxidize reduced sulfur compounds (e.g., thiosulfate, hydrogen sulfide) while simultaneously reducing nitrate to nitrogen oxides or dinitrogen gas. This metabolism is crucial in both natural and anthropogenic systems, including groundwater, sediments, and bioreactors for nitrate and sulfide removal [2].

In addition, *T. denitrificans* has been widely applied in bioaugmentation strategies for the remediation of nitrate- and sulfide-contaminated environments [3,4]. Potential applications include combined removal of sulfur and nitrate compounds from water environments [5] or through activated [6] and anaerobic sludge enrichment [7]. These applications underscore the practical need for accessible and reproducible cultivation protocols suitable for routine laboratory and engineering practice [2,4].

Previous studies have reported cultivation of *T. denitrificans* and *S. denitrificans* using a variety of mineral media and strictly controlled anaerobic or microaerobic cultivation systems, depending on the objectives of the study. Cultivation of *T. denitrificans* generally relies on controlled anaerobic techniques, defined mineral media, and supplementation with essential trace elements to support sulfur oxidation and denitrification metabolism [1,8,9]. Depending on the experimental purpose, growth is evaluated in batch or continuous systems, and enrichment approaches are used, with cultivation conditions optimized for nitrate reduction, sulfur conversion, or biomass production [7,10,11]. In comparison, *S. denitrificans* has mainly been investigated as a model sulfur-oxidizer, nitrate-reducing bacterium involved in marine and environmental redox processes. Cultivation of *S. denitrificans* is typically performed under anaerobic or microaerobic conditions using mineral media supplemented with reduced sulfur compounds or alternative electron donors, with careful control of oxygen availability [8,9].

However, cultivation of these bacteria under laboratory conditions, especially for inexperienced staff, is challenging, particularly for laboratories lacking advanced anaerobic systems. According to the DSMZ (German Collection of Microorganisms and Cell Cultures), *T. denitrificans* and *S. denitrificans* are cultured in specific anoxic conditions and mineral-defined media. Consequently, detailed protocols are scattered across primary literature, DSMZ medium sheets, and supplementary materials and are frequently optimized for specific experimental purposes (e.g., genomics, isotope labeling studies, denitrification kinetics) rather than routine cultivation [8,9,10,11].

From a practical standpoint, establishing working cultures of *T. denitrificans* and *S. denitrificans* without advanced anaerobic infrastructure is challenging and time-consuming. Our laboratory faced this challenge when attempting to establish pure cultures from DSMZ stock strains. It required several months of systematic optimization to develop reproducible cultivation protocols, echoing the quote from the DSMZ guidelines for anaerobic cultivation (DSMZ)—“*However, please keep in mind, that even if described in detail, some steps of the handling of anaerobic cultures are frequently difficult to master without demonstration. For beginners in anaerobic microbiology it is therefore the best to visit a laboratory where anaerobic cultivation techniques are routinely in use.*”

Therefore, this practical report aims to provide systematic and simplified cultivation protocols and systematic troubleshooting guidance for laboratories with limited resources or experience in anaerobic microbiology. We report successful cultivation of *T. denitrificans* under both conventional anaerobic jar conditions and simplified “pseudo-anoxic” conditions. What we provisionally termed “pseudo-anoxic conditions” refer to tightly sealed culture bottles without headspace exchange or reducing agents. The findings demonstrated that successful laboratory cultivation of these organisms does not necessarily require elaborate anaerobic systems, and that historical literature contains valuable practical guidance often overlooked in contemporary keyword-driven searches [10,11].

## 2. Materials and Methods

### 2.1. Bacterial Strains and Inocula

**Figure 1:**
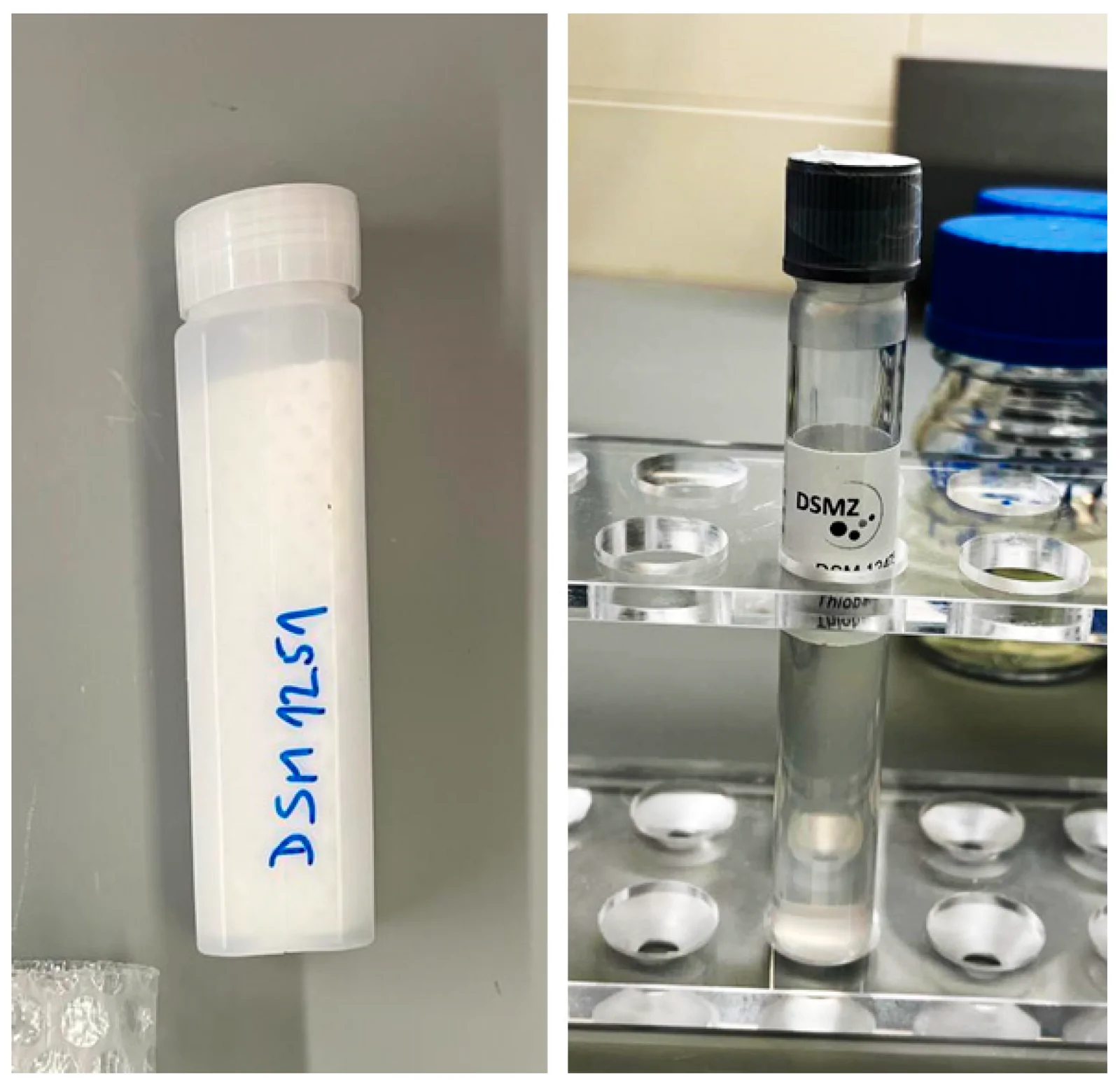
Sealed tubes with actively growing bacterial cultures of *Thiobacillus denitrificans* DSM 12475 and *Sulfurimonas denitrificans* DSM 1251 obtained from DSMZ.

*Thiobacillus denitrificans* DSM 12475 and *Sulfurimonas denitrificans* DSM 1251 were purchased from Deutsche Sammlung von Mikroorganismen und Zellkulturen (DSMZ, Braunschweig, Germany) as actively growing liquid cultures in sealed Hungate glass tubes (Figure 1). Cultures were received in an actively growing state with visible turbidity in DSMZ Medium 113. For inoculation, the suspension was extracted using a sterile syringe and needle under aseptic conditions. From the syringe, the suspension was transferred to culture media. During the transfer, we did not use a specific protocol for anaerobe cultivation (i.e., Hungate technique) as suggested by the DSM guidelines (DSMZ).

### 2.2. Growth Media

Three media were tested in both liquid and solid forms and are designated Media A, B, and C. Complete recipes and instructions for preparation are provided below.

Medium A: Modified DSMZ 113

Medium A was a slightly modified standard DSMZ 113 medium recommended for cultivation of both *T. denitrificans* and *S. denitrificans* (DSMZ). The modification consisted of substituting the trace element solution SL-4 with tap water (see Table 1 for complete composition) originating from Zagreb’s municipal drinking water supply and represents potable drinking water. This substitution was made to simplify medium preparation in laboratories without trace element stock solutions while maintaining essential micronutrient availability through the tap water supply. The hardness of Zagreb water is typically 200–400 mg CaCO_3_/L, and the water contains naturally occurring trace elements [12].

Medium preparation followed this procedure: four separate solutions were prepared as described in Table 1.

Solutions a and c were sterilized by autoclaving at 121 °C for 20 min in a standard laboratory autoclave (not under an N_2_ atmosphere, as listed in the DSMZ protocol). Solutions b and d were sterilized by syringe filtration through a 0.2 µm nitrocellulose filter. The pH of solution a was adjusted to 6.4 prior to autoclaving; other solutions were not pH-adjusted. It is critical to adjust the pH of solution a to 6.4 or above, as otherwise bacteria will not grow. Solutions were then combined in a sterilized laboratory bottle in the following order and proportions: 960 mL of solution a, 2% of solution b, 2% of solution c and 0.1% of solution d.

Solid Medium A was prepared by adding 15 g/L of bacteriological agar (Biolife, Milan, Italy) to solution a prior to autoclaving. After sterilization, solutions b, c and d were added to agar media cooled to 60 °C and poured into Petri dishes.

Later on, as a control, liquid Medium A was also prepared using the standard DSMZ trace element solution (SL-4) instead of tap water, while all other cultivation conditions remained unchanged.

Medium B: ATCC 152 *Thiobacillus* broth

Medium B was an ATCC 152 *Thiobacillus* broth (ATCC—American Type Culture Collection), which is recommended for cultivation of *Thiobacillus* intermedia. While ATCC does not recommend this medium specifically for *T. denitrificans* (ATCC medium 450 T2, which is essentially identical to DSMZ 113, is preferred), we tested Medium B to evaluate a more readily available and simpler-to-prepare alternative that might support growth. The complete formulation is given in Table 2. The medium was autoclaved at 121 °C for 20 min.

Medium C: commercially available *Thiobacillus* broth

Medium C (Table 3) was a commercially available Thiobacillus broth (HIMedia, Mumbai, India), selected to represent media available through standard laboratory suppliers without specialty preparation. Media was autoclaved at 121 °C for 20 min.

### 2.3. Culture Conditions and Anaerobic Systems

During the experimental phase, our primary objective was to establish reproducible growth of the cultures obtained from DSMZ. We tested multiple combinations, including three different media, both anaerobic and aerobic setups, at room temperature (23–25 °C), at 30 °C and at 37 °C.

Anaerobic cultivation using anaerobic jars

True anaerobic conditions were achieved by incubating culture bottles in anaerobic jars using either Anaerocult A^TM^ or Anaerocult C^TM^ reagents (Merck KGaA, Darmstadt, Germany). Culture bottles (80 mL total capacity, containing 50 mL medium) were placed loosely capped inside the jars to ensure the anaerobic atmosphere reached the culture bottles. No additional colorimetric indicator was included, as the validated Anaerocult cultivation system was used to generate anaerobic conditions.

Pseudo-anaerobic cultivation (sealed-bottle method)

In contrast, the second setup represented a pseudo-anoxic environment: after inoculation with 0.5 mL of bacterial suspension, 80 mL bottles containing 50 mL of culture medium were immediately tightly capped with screw caps. Bottles were then incubated statically (no agitation) for 7 days without further disturbance. This simplified cultivation approach was intended to promote depletion of dissolved oxygen within the sealed bottles during static incubation. However, oxygen concentration was not directly monitored using a redox indicator or other oxygen-measuring method.

Growth monitoring and temperature

In all cases, growth was monitored visually by observing culture turbidity. Cultures were incubated at:

- Room temperature (22–25 °C);
- Optimized denitrifier temperature (30 °C);
- Mesophilic temperature (37 °C).

All incubations were conducted in static (non-shaken) conditions for 7 days.

### 2.4. Growth Kinetics

Kinetics of *T. denitrificans* and *S. denitrificans* growth were monitored spectrophotometrically (Hach DR2500 Spectrophotometer, Düsseldorf, Germany) by measuring optical density at a 625 nm wavelength. The spectrometer cuvettes (HACH Cat. No. 2401906, Düsseldorf, Germany) were pre-autoclaved, and 25 mL of Medium A was aseptically added. Next, 100 µL of actively growing bacterial suspension (inoculum) was added. Slightly opened cuvettes were incubated in anaerobic jars with Anaerocult C reagent under pseudo-anaerobic conditions at room temperature (22–25 °C) and at 30 °C for 7 days. The measurement schedule was: once per day for days 1–3, and multiple times per day thereafter (days 4–7). Fresh Anaerocult C catalyst was added after each measurement to maintain anaerobic conditions. Optical density was measured against non-inoculated Medium A, which served as the blank for all spectrophotometric measurements.

Three biological replicates were prepared for each cultivation condition. Data are presented as mean values ± standard deviation.

### 2.5. Microscopy for Colony Morphology and Motility Assessment

Microscopic examinations were performed on a Bresser Optik 5760700 Science Infinity microscope (Bresser, Rhede, Germany) equipped with a C-mount RisingCam^®^ (Hangzhou, China) digital microscope camera with a Sony IMX678 CMOS sensor (8.4 MP, 1/1.8″, 2.0 µm pixel size, Minato, Japan) for imaging.

Gram staining of colonies and pure cultures was performed using standard protocols and observed at 1000× magnification to assess cell morphology and Gram reaction.

Additionally, a novel, simple method for direct observation of colony morphology and bacterial motility was applied. The procedure was as follows: agar plates containing suspected *T. denitrificans* colonies (typically very small, transparent, barely visible to the naked eye) grown under anaerobic conditions using Medium A were used, and a glass coverslip was gently placed directly on the agar surface over the area of interest. The plate was then placed on a standard microscope stage and examined at 40× and 100× magnification. Next, a drop of immersion oil was placed on the cover slip, and an immersion objective was used (1000× magnification). This method allowed for rapid, non-destructive assessment of colony morphology and cell motility (via characteristic twitching movements of motile rod-shaped cells) without removing colonies from the agar or adding stains.

## 3. Results

### 3.1. Comparative Growth in Liquid Media

**Figure 2:**
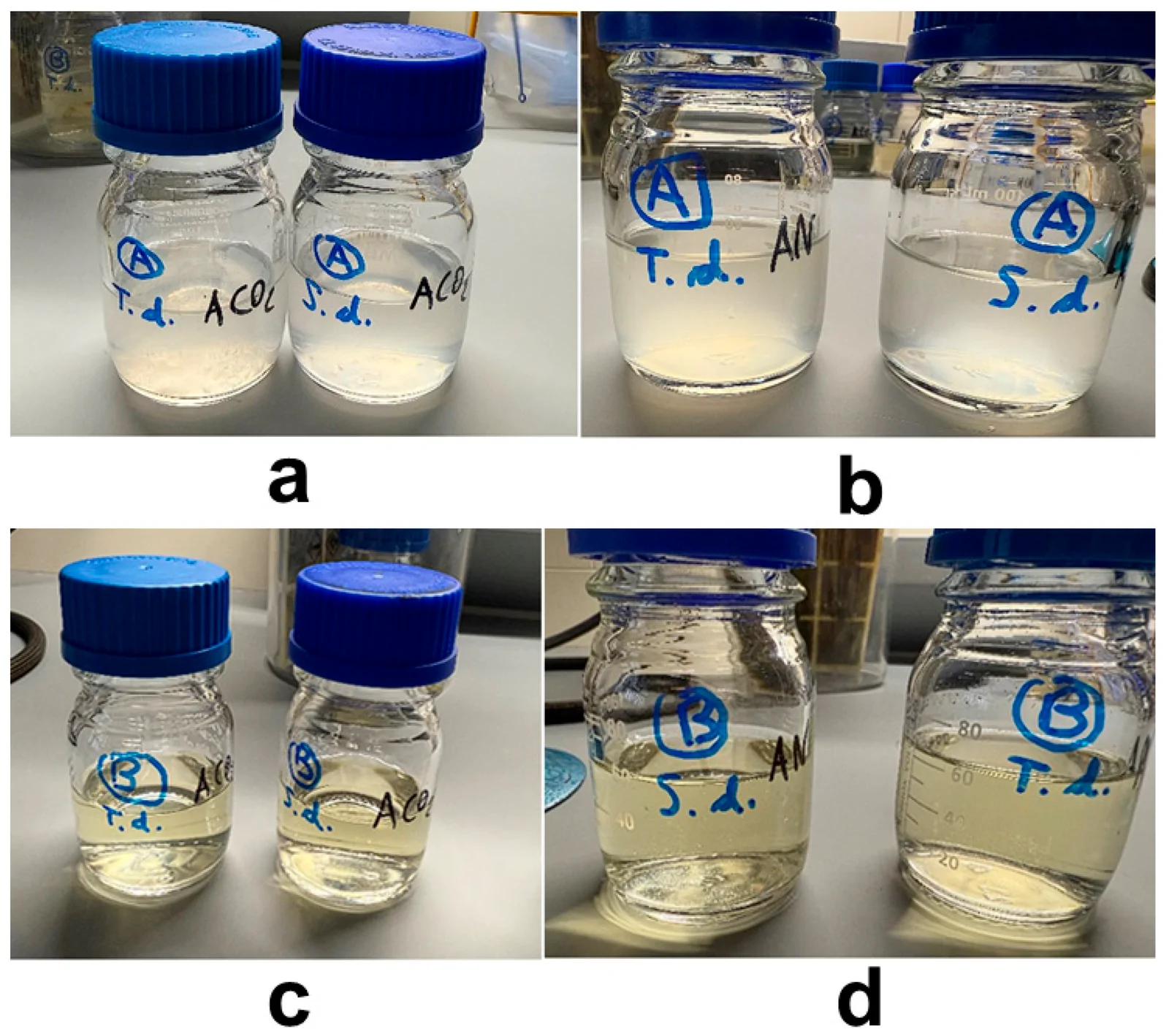
Turbidity of bottles containing *Thiobacillus denitrificans* (T.d.) and *Sulfurimonas denitrificans* (S.d.) in Medium A (**a**,**b**), and transparent bottles with Medium B (**c**,**d**), after one week of incubation at room temperature in anaerobic jars with Anaerocult™ C (**a**,**c**) and Anaerocult™ A (**b**,**d**).

Among the three media tested, only Medium A (modified DSMZ 113) supported observable growth of both *T. denitrificans* and *S. denitrificans* (Figure 2). No growth was achieved in Medium B (ATCC 152) and Medium C (commercial *Thiobacillus* broth) after 7 days of incubation, regardless of anoxic conditions or temperature.

*T. denitrificans* grew successfully in Medium A under both true anaerobic jar conditions (using Anaerocult A or C) and pseudo-anaerobic sealed-bottle conditions. Visually, growth appeared comparable between the two approaches. Both cultivation methods supported growth at room temperature (22–25 °C) and at 30 °C, but growth was not observed at 37 °C in any case (Table 4).

*S. denitrificans* grew reliably in Medium A only under true anaerobic jar conditions (Anaerocult A or C). No growth was observed under pseudo-anoxic sealed-bottle conditions (Table 4), suggesting that this organism requires a more strictly controlled anoxic environment than *T. denitrificans* Additionally, some subcultures of *S. denitrificans* occasionally failed to grow despite proper technique and favorable conditions, suggesting metabolic sensitivity to subtle culture variations.

As a positive control, comparable growth of both bacteria was obtained when Medium A was prepared with the standard DSMZ trace element solution (SL-4) instead of tap water, confirming that the simplified cultivation protocol is not dependent on the composition of the local tap water supply.

### 3.2. Growth on Solid Media

After many attempts, the conclusion is that observable growth on solid media (colonies visible with naked eye) occurred only on Medium A with addition of agar, using Anaerocult, for *T. denitrificans*. *S. denitrificans* was not observed to grow on solid medium. Subculturing (solid media to solid media) was also possible in the case of *T. denitrificans*.

**Figure 3:**
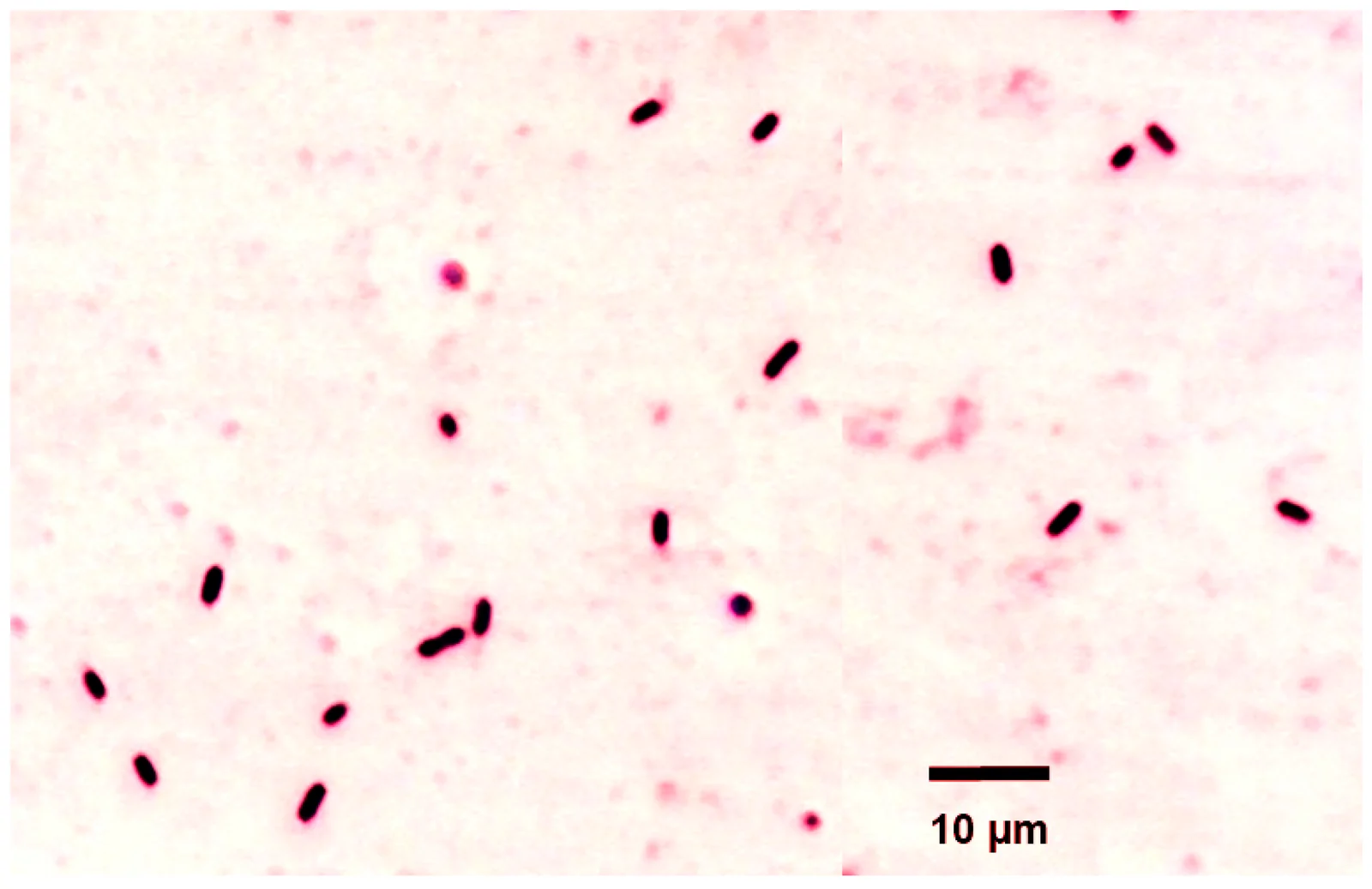
Gram stain of *T. denitrificans*. Imaged by C-mount RisingCam^®^ digital microscope camera equipped with a Sony IMX678 CMOS sensor (8.4 MP, 1/1.8″, 2.0 µm pixel size).

Several practical observations should be listed here. Initially, we were convinced that *T. denitrificans* did not grow on solid plates, as no colonies could be clearly seen with the naked eye after one week of incubation. On one occasion, there was a clear case of contamination on the plates inoculated with *T. denitrificans*, but Gram-stained microscopy slides revealed that in addition to contamination, many cells of *T. denitrificans* were present on the plate—*T. denitrificans* has a distinguishable shape of a very thin, short and elegant rod (Figure 3).

**Figure 4:**
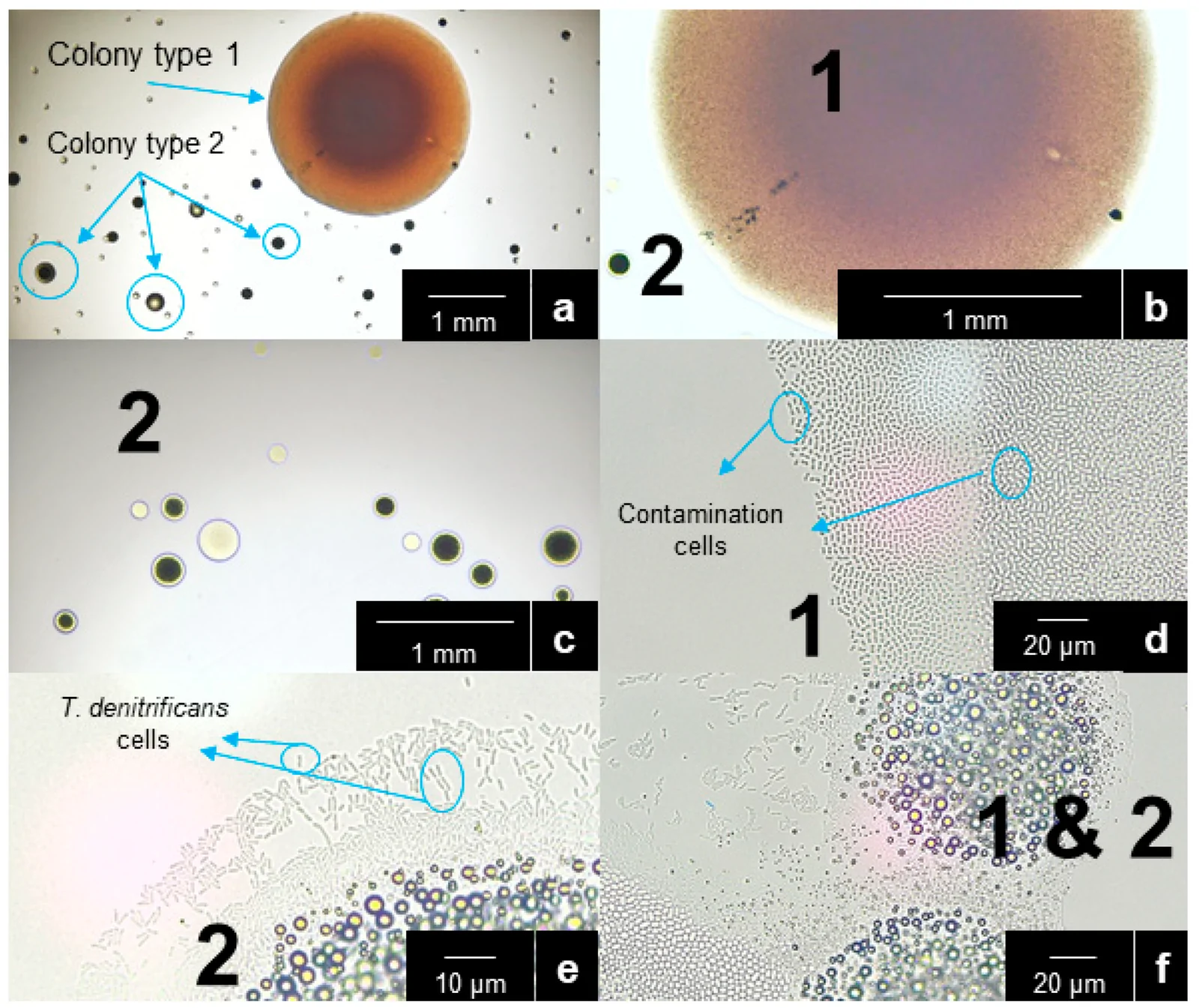
Microscopic analysis of mixed colonies on agar. (**a**) Overview of the plate at 40× magnification showing large brown colonies (type 1) and numerous tiny, transparent colonies (type 2). (**b**,**c**) Higher magnification (100×) confirms the presence of two distinct colony morphotypes, but their identity cannot be resolved. (**d**) Visualization of the large colony type using the coverslip-on-agar immersion method reveals non-motile, thick rod-shaped bacterial cells, consistent with a contaminant. (**e**) The tiny, transparent colonies consist of thin rod-shaped, clearly motile cells typical of *Thiobacillus denitrificans*. (**f**) Field showing both cell types in close proximity, with motile *T. denitrificans* cells apparently “invading” the static contaminant colony.

After additional careful examination of the plates, we noticed tiny, transparent and almost invisible colonies. The agar plate was mounted on the microscope stand for further examination and examined with the smallest magnification (40×). Indeed, two distinct colony types were noticeable (Figure 4a). We presumed the contamination consisted of larger colonies, but at this magnification, it was impossible to differentiate which was the contamination from possible *T. denitrificans* colonies. Using a larger magnification (100×) confirmed two types of colonies (Figure 4b), but still no conclusion could be made (Figure 4c). We were reluctant to use higher magnification objectives to avoid contacting the colonies.

Therefore, we applied a simple coverslip-on-agar approach that allowed direct microscopic observation of the colonies. Although similar practical approaches may occasionally be used informally in microbiology laboratories, we were unable to find a description of this specific application in the literature. Basically, a cover slide was placed on the agar surface and then viewed using immersion oil. Surprisingly, this method functioned perfectly, and we were able to clearly observe two morphologically different types of cells. The suspected contamination consisted of bacterial cells with a thick rod shape that were non-motile (Figure 4d). In contrast, bacterial cells of tiny colonies were shaped like thin rods, a shape characteristic of *T. denitrificans*, and clearly motile (Figure 4e). It was interesting to observe both cell types next to one another and motile cells of *T. denitrificans* that seem to be “invading” the static colony (Figure 4f). This microscopy technique opened possibilities for rapid confirmation of bacterial morphology and motility, the latter being advantageous relative to classic Gram staining.

**Figure 5:**
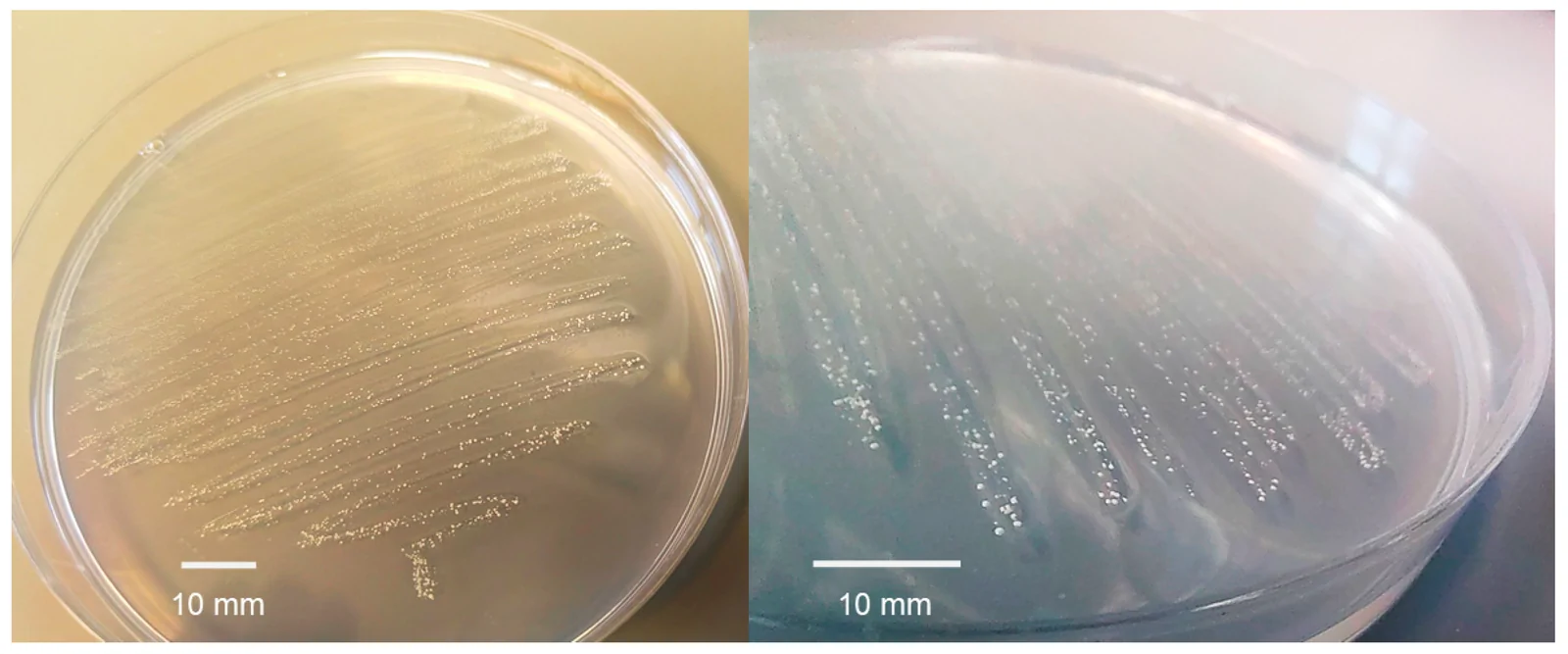
Pure culture of *T. denitrificans* cultivated on Medium A agar plates after 14 days of incubation at room temperature in AnaerocultC^TM^.

Later on, a pure culture of *T. denitrificans* was successfully subcultured onto fresh Medium A agar plates, producing tiny colonies after approximately 14 days of incubation (Figure 5).

### 3.3. Culture Storage and Subculturing

**Figure 6:**
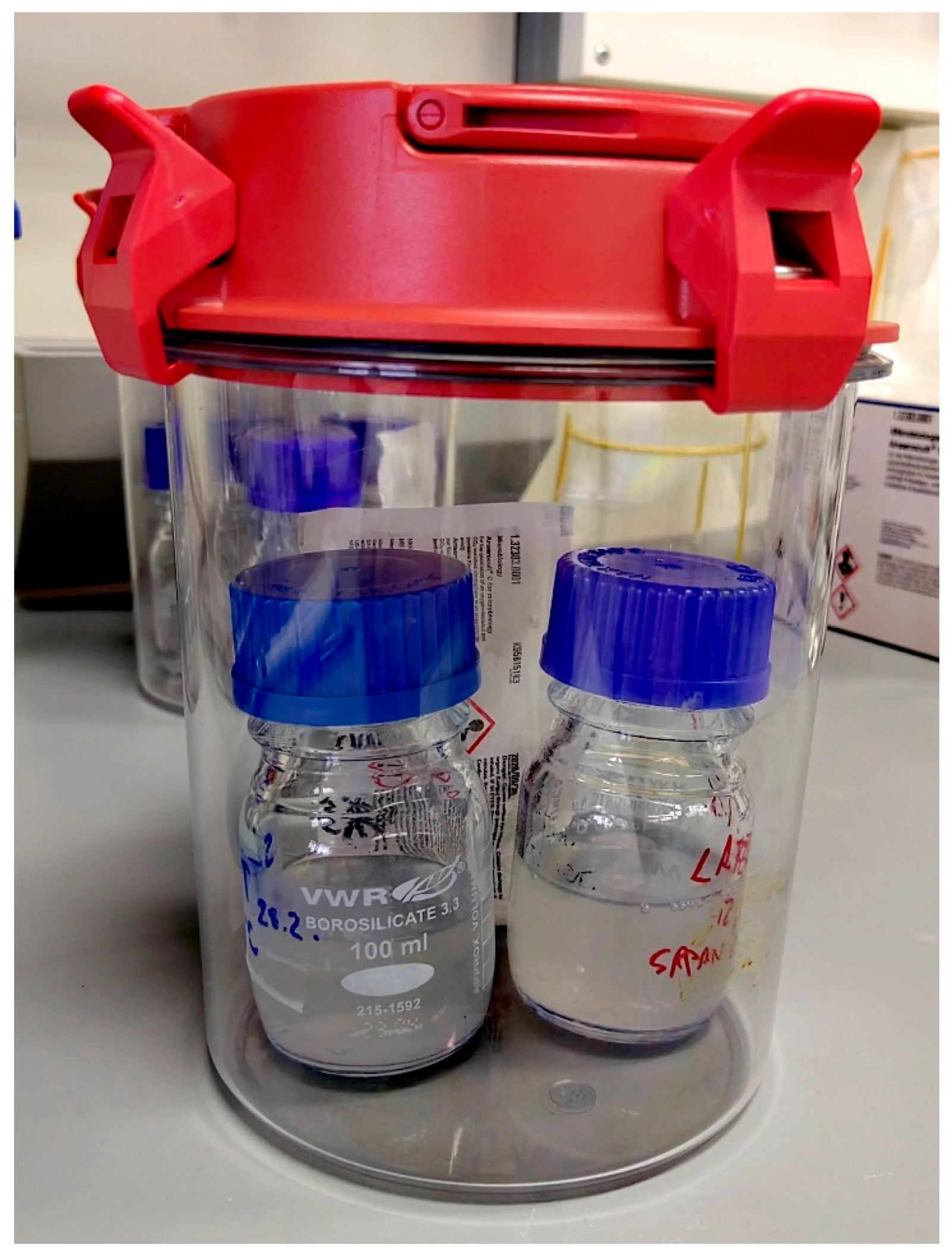
Storage of active cultures of *T. denitrificans* and *S. denitrificans* in Anaerocult.

Both *T. denitrificans* and *S. denitrificans* were best maintained in liquid Medium A in anaerobic jars with AnaerocultC^TM^ at 4 °C (refrigerated conditions) (Figure 6). Even after several months, subculturing from these stored cultures yielded positive growth. Successive subculturing for several passages was also performed without any problems, but only with *T. denitrificans*. In the case of *T. denitrificans*, all of the aforementioned observations were also applicable in pseudo-anoxic conditions; storage, subculturing and successive subculturing all yielded positive growth.

### 3.4. Growth Kinetics of T. denitrificans

**Figure 7:**
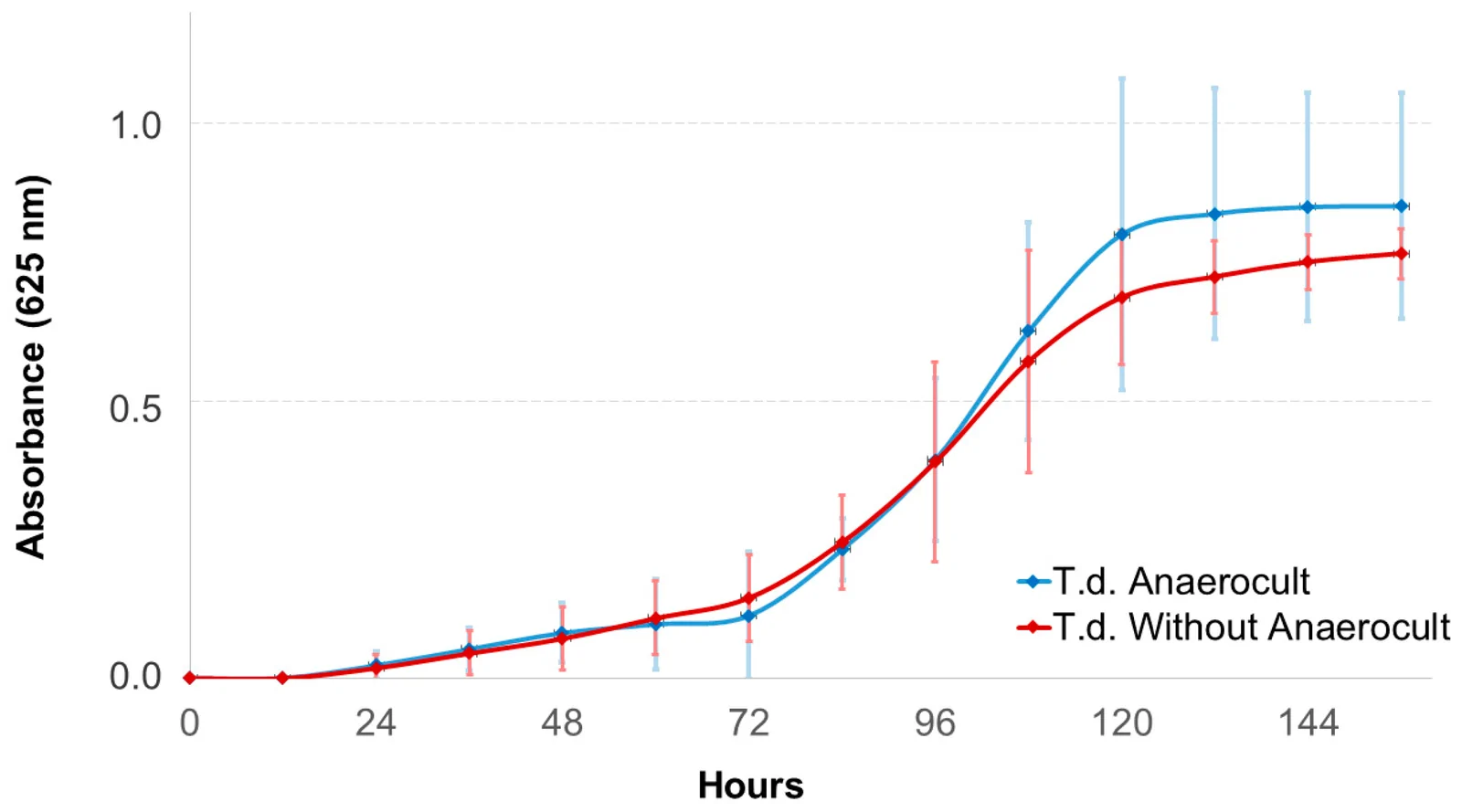
Growth kinetics of *T. denitrificans* at 22–25 °C, measured by optical density (OD_625_) and incubated with or without the use of Anaerocult. Data are presented as mean values ± standard deviations from three biological replicates.

*T. denitrificans* exhibited characteristic Monodian growth kinetics (Figure 7). The culture showed a prolonged lag phase (0–72 h), followed by exponential growth (approximately days 3–5), and entry into stationary (lag) phase between days 5–7. An incubation period of approximately 7 days at room temperature or 30 °C was therefore sufficient to reach the stationary phase.

**Figure 8:**
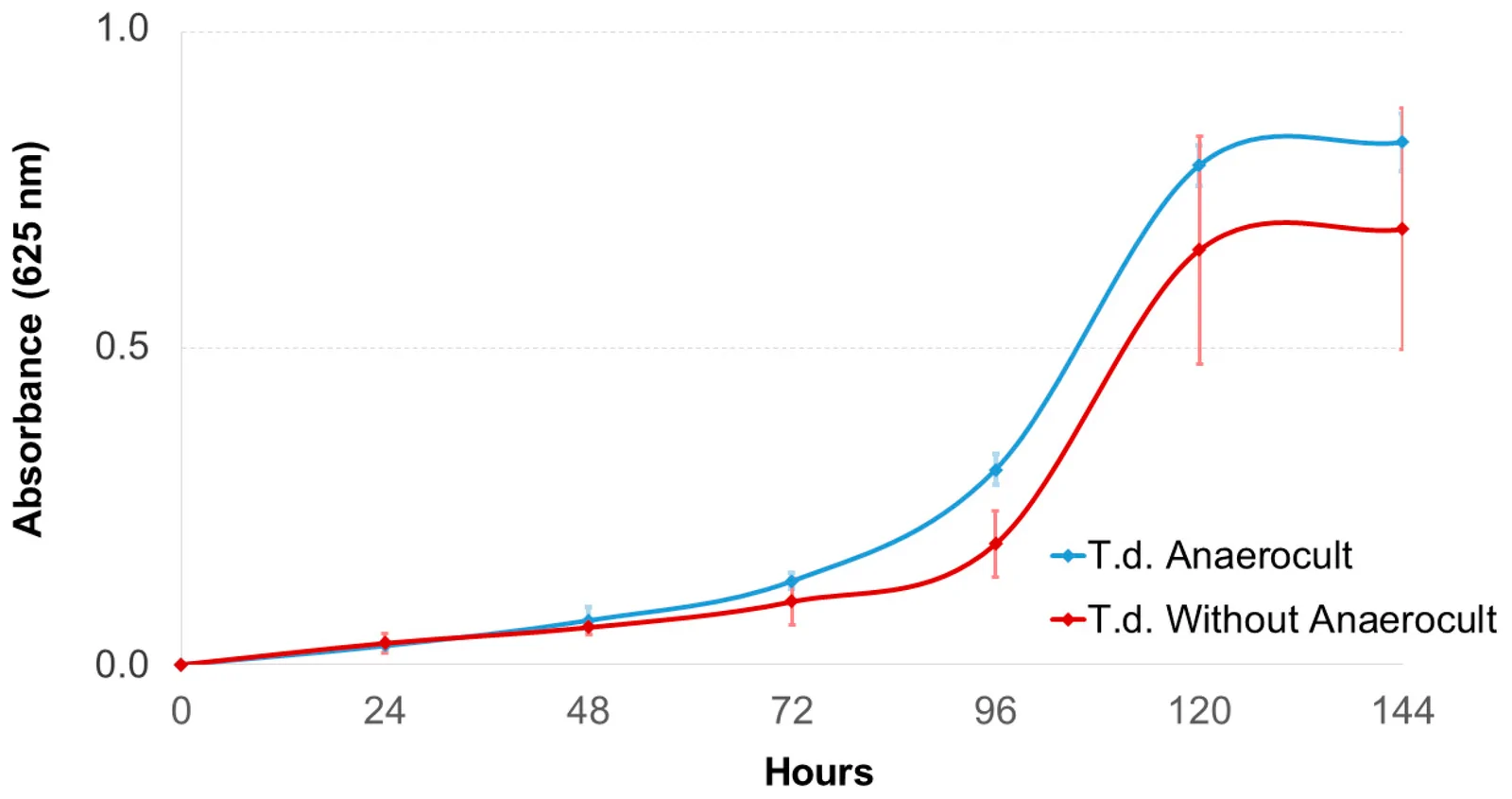
Growth kinetics of *T. denitrificans* at 30 °C, measured by optical density (OD_625_) and incubated with or without the use of Anaerocult. Data are presented as mean values ± standard deviations from three biological replicates.

At both 22–25 °C and 30 °C, exponential growth commenced at approximately 72 h post-inoculation. However, growth at 30 °C proceeded with a seemingly steeper exponential slope, indicating faster growth kinetics (Figure 7 and Figure 8). In contrast, growth at 22–25 °C was slower in terms of growth rate, but cultures achieved comparable or equivalent stationary-phase biomass (final OD_625_ values) by day 7 (Figure 7 and Figure 8).

There was no significant difference in growth profile between cultures incubated with or without Anaerocult catalyst at either temperature, indicating that growth is essentially equivalent under true anaerobic jar conditions and pseudo-anaerobic sealed-bottle setup for this organism.

### 3.5. Comparative Cultivation: T. denitrificans vs. S. denitrificans

From a practical standpoint, *T. denitrificans* was considerably easier to cultivate reliably than *S. denitrificans*. Growth of *S. denitrificans* under anaerobic jar conditions confirmed culture viability and served as a positive control; however, no growth was observed under pseudo-anoxic conditions, likely reflecting the stricter oxygen sensitivity of this organism. In addition, *S. denitrificans* consistently produced lower biomass across all tested conditions and occasionally failed to resume growth after subculturing despite proper aseptic technique and anoxic incubation. In contrast, *T. denitrificans* produced robust growth across a wider range of conditions, including pseudo-anaerobic sealed bottles, reached higher final biomass, and showed reliable subculturing success.

## 4. Discussion

### 4.1. Simplified and Accessible Cultivation Protocols for Chemolithoautotrophs

The enhanced growth yield observed in Medium A can be attributed to its richer nutrient composition tailored for obligate chemolithoautotrophs [1,13]. Compared to Medium B and Medium C, Medium A provides a higher availability of inorganic carbon (HCO_3_^−^/CO_2_) for autotrophic carbon fixation, nitrates (NO_3_^−^) to serve as terminal electron acceptors for anaerobic respiration, and essential trace metals serving as cofactors for key metabolic enzymes. Additionally, its superior buffering capacity prevents premature growth arrest caused by metabolic acidification [13].

When microbiologists encounter novel cultivation methods or attempt to grow microorganisms outside routine laboratory practice, they face significant practical challenges: How is this organism actually cultured? These days, a usual first step in new research is a query to an internet search engine or an AI-based tool, which typically yields the recommendation that these organisms can only be cultivated under a strictly controlled anaerobic setup, often with detailed gas-mixing protocols and specialized equipment requirements.

Protocols in most cases do exist, but they are frequently scattered across the literature, adapted for specific experimental purposes, and presented in ways that appear complex or even unfeasible without specialized equipment. For sulfur-oxidizing, nitrate-reducing bacteria, such as *T. denitrificans* and *S. denitrificans*, this represents a significant obstacle for laboratories lacking access to advanced anaerobic systems (anaerobic incubators, gas-mixing systems, Hungate tube techniques), which are frequently considered indispensable for the cultivation of such organisms [14,15]. Recent papers report on the cultivation of both bacteria under strictly anaerobic conditions in a CO_2_/N_2_ gas atmosphere. Beller et al. [1] cultivated *T. denitrificans* at 30 °C under strictly anaerobic conditions, and Becker et al. [16] investigated *T. denitrificans* ATCC 25259 under nitrate-reducing, Fe(II)-oxidizing conditions using strictly anaerobic protocols at 30 °C. Another approach for the cultivation of anaerobic bacteria is the addition of a reducing agent, which has shown potential to substitute fully anaerobic setups and to allow growth of certain anaerobic bacteria without anoxic conditions [17].

However, our findings suggest a more nuanced picture. *T. denitrificans* was successfully and reproducibly cultivated under two distinct operational regimes: true anoxic conditions using standard anaerobic jars (Anaerocult™ systems) with professional-grade control of atmospheric oxygen, and under pseudo-anoxic conditions using simple, tightly sealed bottles without any specialized anaerobic apparatus or gas management.

Interestingly, this observation is not entirely novel. Historical literature, including the pioneering work of Justin & Kelly [10] and more recent reports by Zhang et al. [11], also reported successful cultivation outside of strictly anaerobic setups, demonstrating metabolic flexibility.

However, such practical guidance is currently difficult to retrieve through keyword-driven searches and AI interfaces, which tend to emphasize strictly anaerobic, chamber-based approaches. This raises the question of why older yet highly relevant experimental insights have been marginalized in the era of modern search engines and keyword-driven literature retrieval. The neglect of such knowledge not only complicates experimental reproducibility but also reinforces a misconception that certain organisms are more difficult, if not impossible, to cultivate than they truly are.

Most studies cultivate *T. denitrificans* at 30 °C to maximize growth rates; however, room-temperature cultivation was selected in our research to evaluate the robustness of growth under conditions more representative of routine laboratory handling. Despite slower exponential growth, cultures reached similar stationary-phase biomass, suggesting that *T. denitrificans* tolerates suboptimal temperatures without a substantial loss in growth capacity. This observation may be particularly relevant for larger-scale cultivation setups, where maintaining ambient temperatures could facilitate simpler and more energy-efficient growth conditions.

*S. denitrificans* was included to assess whether the simplified cultivation approach could be extended beyond a single model organism. The contrasting outcomes observed here proved informative, as they demonstrate that while *T. denitrificans* can be reliably cultivated under simplified pseudo-anoxic conditions, this cannot be generalized to all chemolithoautotrophic denitrifiers. In this regard, the requirement of *S. denitrificans* for a strictly anaerobic setup highlights the species-specific nature of cultivation constraints and underscores the importance of testing simplified protocols across multiple organisms.

### 4.2. Medium Optimization: Tap Water Substitution and Reproducibility

The substitution of trace element solution SL-4 with tap water in Medium A was undertaken to simplify medium preparation in resource-limited settings. While this modification was successful in our hands (supporting robust growth comparable to the standard formulation), it introduces a critical caveat: the exact composition of tap water is inherently site-dependent and varies by geographic location and water treatment practices.

Our local tap water (potable, Zagreb public water supply) contains hardness in the range of 200–400 mg CaCO_3_/L and contains naturally occurring trace elements, including iron, zinc, and manganese [12]. These elements, among others, present at trace concentrations, may have contributed to the observed growth support. However, the individual element(s) responsible for this effect cannot be identified without detailed chemical characterization of the water composition.

However, this approach may not be universally reproducible across different laboratories or geographic regions with different water chemistries. Because the observed growth was not dependent on specific components of the local tap water, additional control experiments were performed using the standard trace element solution. Comparable growth of both *T. denitrificans* and *S. denitrificans* was observed under these conditions, confirming that the simplified cultivation protocol is not reliant on the particular composition of the local tap water supply. However, laboratories aiming for maximum reproducibility and consistency are strongly advised to use defined trace element solutions (e.g., DSMZ SL-4 solution as specified in the original DSMZ 113 medium) rather than relying on tap water composition.

Tap water substitution is presented here as a practical alternative for initial cultivation and maintenance of cultures in resource-limited settings, with the explicit understanding that growth performance and consistency may vary with local water composition. This limitation does not negate the utility of the simplified protocol but rather emphasizes the importance of quality control and potential medium adjustments for laboratories in different geographic locations.

### 4.3. Growth at Room Temperature vs. Optimized Denitrifier Temperature

Most studies in the literature optimize cultivation of *T. denitrificans* at 30 °C to maximize growth rate, as this is considered its optimum [10,18,19]. Our results demonstrate that at room temperature, the cultures reached stationary-phase biomass comparable to that obtained at 30 °C after seven days of incubation. This observation has practical implications for larger-scale or longer-term cultivation, where room-temperature operation could enable simpler and more energy-efficient protocols without substantial loss of final biomass yield. Growth at 37 °C was tested to evaluate the potential use of *T. denitrificans* for direct bioaugmentation in mesophilic reactor systems; however, no growth was observed at this temperature.

### 4.4. Coverslip-on-Agar Microscopy as a Rapid Approach for Colony Characterization

The coverslip-on-agar approach described here represents a methodologically simple technique for direct observation of colony morphology and assessment of bacterial motility without removing colonies from the agar surface or applying stains. The method exploits the fact that bacteria with characteristic morphologies and motility can be readily distinguished from contaminants under classic optical microscopy via a coverslip.

This approach is particularly valuable for bacteria that form very small, slow-growing, or nearly transparent colonies (such as *T. denitrificans*), which are easily overlooked on routine microscopic inspection. The direct visualization of motility is a bonus advantage, as motility is an excellent phenotypic marker for many bacterial species.

To the best of our knowledge, this specific application of the coverslip method for plate colony examination has not been formally described in the literature. It is not, however, excluded that similar practical approaches are perhaps part of informal laboratory practice and may be transmitted through hands-on training and direct communication between microbiologists. The technique is robust, requires no special equipment beyond a standard microscope, and can serve as a rapid, non-destructive confirmation of colony identity and purity, making it particularly useful for teaching laboratories and resource-limited settings.

## 5. Conclusions

Successful laboratory cultivation of *Thiobacillus denitrificans* and *Sulfurimonas denitrificans* is achievable in resource-limited settings using accessible equipment and simplified protocols. *T. denitrificans* grows robustly in modified DSMZ 113 medium under both true anaerobic (Anaerocult jar) and pseudo-anaerobic (tightly sealed bottle) setups, with comparable growth kinetics and stationary-phase biomass at room temperature (22–25 °C) and at 30 °C. Turbidity became clearly visible after 5–7 days. Growth was consistent and reproducible at room temperature (22–25 °C) and at 30 °C, but was absent at 37 °C. In contrast, *S. denitrificans* requires true anaerobic jar conditions (Anaerocult) to be reliably cultured using the simplified DSMZ 113-based medium at 22–25 °C or 30 °C for 7 days. *T. denitrificans* could also be reliably cultivated on solid media, but only in anoxic conditions, producing tiny, transparent, and easily overlooked colonies. The simple coverslip-on-agar microscopy technique enabled rapid discrimination of *T. denitrificans* colonies without specialized equipment or dying, facilitating identification and assessment of motility.

For short-term storage, cultures were kept refrigerated (4 °C) in loosely screwed bottles placed inside an Anaerocult™ jar. Under these conditions, both species remained viable for weeks; however, after approximately five months we were unable to revive cultures from these stocks. This highlights that such storage is only suitable as a temporary solution. For optimal maintenance, cultures should be re-passaged at least once per month under optimal growth conditions to ensure sustained viability and prevent loss of cultivability.

These findings demonstrate that reproducible, accessible cultivation protocols for certain chemolithoautotrophic denitrifiers do not require elaborate anaerobic infrastructure. Rather, systematic optimization of medium composition, temperature, and operational protocols can overcome many of the practical barriers to successful laboratory cultivation. This work provides step-by-step guidance suitable for teaching laboratories, startup facilities, and research groups in resource-limited settings seeking to establish or maintain cultures of *T. denitrificans* or *S. denitrificans* for bioaugmentation, bioreactor seed development, or academic research.

## Figures and Tables

**Table 1:** Recipe for Medium A (DSMZ 113 medium). Modifications are noted in the Table.

**DSMZ_113 Medium**	
**Solution *a***	
KH_2_PO_4_	2 g
KNO_3_	2 g
NH_4_Cl	1 g
MgSO_4_ × 7 H_2_O	0.8 g
Trace element solution SL-4 (**instead, we added 20 mL of tap water**)	2 mL
Distilled water (**we added 940 mL, so total volume was 960 mL**)	960 mL
**Solution *b***	
Na_2_S_2_O_3_ × 5 H_2_O	5 g
Distilled water	20 mL
**Solution *c***	
Na_2_CO_3_	1 g
Distilled water	20 mL
**Solution *d***	
FeSO_4_ × 7 H_2_O	20 mg
H_2_SO_4_ (0.1 N)	10 mL
Trace element solution SL-4	
Na_2_-EDTA	0.5 g
FeSO_4_ × 7 H_2_O	0.2 g
ZnSO_4_ × 7 H_2_O	0.1 g
MnCl_2_ × 4 H_2_O	0.03 g
H_3_BO_3_	0.3 g
CoCl_2_ × 6 H_2_O	0.2 g
CuCl_2_ × 2 H_2_O	0.01 g
NiCl_2_ × 6 H_2_O	0.02 g
Na_2_MoO_4_ × 2 H_2_O	0.03 g
Distilled water	1000 mL

**Table 2:** ATCC 152 Thiobacillus broth composition.

Component	Quantity (Per 1 L)
Chlorophenol red	0.08 g
Na_2_S_2_O_3_ × 5 H_2_O	10 g
NH_4_Cl	1 g
MgCl_2_	0.5 g
K_2_HPO_4_	0.6 g
KH_2_PO_4_	0.4 g
FeCl_2_	0.02 g
Yeast extract	1 g
Distilled water	1000 mL

**Table 3:** Composition of commercial *Thiobacillus* broth (HIMedia, India).

Component	Quantity (Per 1 L)
(NH_4_)_2_SO_4_	0.4 g
KH_2_PO_4_	4 g
CaCl_2_	0.25 g
FeSO_4_ × 7 H_2_O	0.01 g
MgSO_4_ × 7 H_2_O	0.5 g
Na_2_S_2_O_3_ × 5 H_2_O	5 g
Distilled water	1000 mL

**Table 4:** Summary of growth outcomes by medium, organism, and cultivation condition. (+) robust growth with visible turbidity after 5–7 days; (–) no visible growth.

Organism	Medium	Temperature (°C)	Anaerobic Cultivation	Pseudo-Anaerobic Cultivation
*T. denitrificans*	A	22–25, 30	+++	+++
*T. denitrificans*	B, C	22–25, 30	–	–
*S. denitrificans*	A	22–25, 30	++	–
*S. denitrificans*	B, C	22–25, 30	–	–
*T. denitrificans* and *S. denitrificans*	A	37	–	–

## Data Availability

The original contributions presented in this study are included in the article. Further inquiries can be directed to the corresponding authors.
